# The Story as a Quality Instrument: Developing an Instrument for Quality Improvement Based on Narratives of Older Adults Receiving Long-Term Care

**DOI:** 10.3390/ijerph18052773

**Published:** 2021-03-09

**Authors:** Aukelien Scheffelaar, Meriam Janssen, Katrien Luijkx

**Affiliations:** Department Tranzo, Tilburg School of Social and Behavioral Sciences, Tilburg University, P.O. Box 90153, 5000 LE Tilburg, The Netherlands; M.Janssen4@tilburguniversity.edu (M.J.); K.G.Luijkx@tilburguniversity.edu (K.L.)

**Keywords:** quality of care, narrative research, long-term care, older adults, client perspective, quality improvement, insider-researcher, nursing home care, person-centered care

## Abstract

The individual experiences of older adults in long-term care are broadly recognized as an important source of information for measuring wellbeing and quality of care. Narrative research is a special type of qualitative research to elicit people’s individual, diverse experiences in the context of their lifeworld. Narratives are potentially useful for long-term care improvement as they can provide a rich description of an older adult’s life from their own point of view, including the provided care. Little is known about how narratives can best be collected and used to stimulate learning and quality improvement in long-term care for older adults. The current study takes a theoretical approach to developing a narrative quality instrument for care practice in order to discover the experiences of older adults receiving long-term care. The new narrative quality instrument is based on the available literature describing narrative research methodology. The instrument is deemed promising for practice, as it allows care professionals to collect narratives among older adults in a thorough manner for team reflection in order to improve the quality of care. In the future, the feasibility and usability of the instrument will have to be empirically tested.

## 1. Introduction

Trends in western societies have increased the attention paid to the individual needs and preferences of persons receiving care and treatment. Holding the values of the person receiving care central in decision making is a central feature of person-centered care [1]. It has been advocated that the primary focus on technical quality be moved towards the experiential elements of care, in which the experiences of clients and their families are seen as an important source [2]. Person-centered care prioritizes the wellbeing and quality of life outcomes of service users and families to enable the stakeholders to flourish in care [3]. Emphasis is placed on the ability of care professionals to view and treat older adults as holistic beings, and to contextualize knowledge in the life-world of older adults [3].

Given their experience, older adults receiving care are legitimately positioned to have a say in that care and its evaluation by determining the extent to which their needs and preferences are being met [4]. Quantitative and qualitative instruments have both been used to study the experiences of older adults as regards the quality of care, although quantitative instruments dominated traditionally. In the Netherlands, the Consumer Quality Index (CQ index) has been embraced as the national standard quantitative survey instrument to increase external transparency in long-term care. A central database allows for national comparisons, benchmarking and public reporting [5]. In 1998 already, Van Campen et al. reported four disadvantages of using quantitative instruments to map patient perceptions of the quality of healthcare. Surveys for quality research often produce highly skewed scores in which the majority of clients appear to be satisfied. Survey scores also do not provide information about individual levels of expectations, needs and wishes. Rather than the client perspective being central, surveys often evaluate services from the supplier’s point of view; for example, by only including the topics relevant for the quality system of care organizations. Finally, the surveys often contain generic items only, without taking into account specific contextual or illness-related attributes [6].

Qualitative instruments, on the other hand, convey more nuance, detail, and emotional content and can provide a nuanced view of a client’s lifeworld [7]. Qualitative data provides a readable and memorable source which professionals can use for reflection on the strengths and weaknesses of the care provided. Qualitative data might make it easier to interpret and identify specific priorities for improvement [8]. National policy and quality documents published in 2014 and 2017 focused on nursing care for older adults in the Netherlands and emphasized the value of qualitative data for reflection in practice. Reflection by, and an open dialogue between, stakeholders is advocated for improving quality in a specific care context. Emphasis is placed on learning as a basis for quality improvement [9,10].

Narrative research is a special type of qualitative research where respondents interpret their own experiences by telling their individual story in their own way to the researcher in an interview. These storylines offer a fresh and humane outlook on the life world of clients [11]. An attempt is made to minimize the influence of the interviewer by avoiding the question-answer structure which is common in structured or semi-structured interviews and by avoiding restructuring, as the researcher does not question the responses of the interviewee but simply encourages the interviewee to tell their story [12]. The researcher then interprets the meaning of the interview through an analysis of the narrative, hereby making a new interpretation [13]. The research findings are therefore a joint product of the participant who shares their experiences and the researcher who analyzes them [14].

Narrative inquiry can capture the experiences of people in care, and provide insights into the vital components of care for each individual. A narrative showing the connectedness of someone’s experiences with a specific care context and their previous experiences can provide a detailed view of the experiences of a person receiving care [15]. Narratives provide a rich description of a person’s experiences and an exploration of the meanings that an individual derives from their experiences. It helps others to understand specific issues, such as quality of care, from the respondent’s point of view within their social context [16]. An open approach in which client experiences are viewed more broadly than through only the clinical pathway might facilitate clients to talk about their experiences more naturally, as clients do not perceive concepts such as quality, safety, and cost as demarcated [17].

Narratives can be used in several ways in a care context, for example, in the professionalization and training of care professionals, quality improvement, to obtain a more detailed clinical picture of an individual client, in the health advocacy and activism of client groups, and in tailoring communication for a specific client [18]. The primary focus of this paper is the use of narratives for quality research. Most qualitative quality instruments developed for Dutch nursing home care are not built on a theoretical foundation or substantiated with evidence regarding validity, reliability and user experiences [19]. The need for a systematic procedure for collecting, analyzing, and synthesizing narratives to stimulate learning and quality improvement is therefore emphasized [20]. Little is known about how narratives can best be collected and used in practice for quality improvement, as use of the narrative method to structurally assess long-term care for older adults is relatively new [21].

The current study takes a theoretical approach to developing and substantiating a quality instrument for practice to discover the experiences of older adults receiving long-term care. The design of a new narrative quality instrument is based on the available literature describing narrative research. The main question is, what theoretical principles and techniques from the literature on narrative research can be used in the design of a new quality instrument to discover the experiences of older adults receiving long-term care? Care professionals will be appointed as insider researchers, and perform a central role in the instrument as interviewers, in order to stimulate learning, increase understanding of the client perspective, benefit from their contextual knowledge, and to create support for the measures emerging from quality research.

## 2. Methods

The literature on narrative research methodology was reviewed in order to define design foundations for the development of a narrative quality instrument. The theoretical principles and techniques of the narrative method were derived from the literature so as to develop and substantiate a narrative quality instrument. A narrative quality instrument was developed for to measure the quality of long-term care provided to older adults with a physical or mental frailty.

### Theoretical Development of a Quality Instrument

Books and articles published up to 10 October 2020 were reviewed. Searches were conducted in the following databases: Catalogus WorldCat Discovery, Google Scholar, CINAHL, MEDLINE and PsycINFO. The search terms used were “narrative research”, “narrative inquiry”, “story-telling”, “narrative analysis”, “narrative”, and were used both on their own and also combined with [AND] “method”, “methodology”, “practice development”, “quality research”, “quality assessment” [OR] “older people”, “health care”, “long-term care”, “nursing care”, “home care”, and “person-centered care”. The search covered academic books and articles published in peer-reviewed journals in English. The resulting articles and books were examined for the relevance of their content in determining the key principles and techniques of the narrative research methodology and, when determined as useful, summarized for the synthesis. The reference lists of the relevant articles were examined to identify further relevant publications that had been missed in the search. Relevant journals were reviewed (e.g., Narrative Inquiry, Qualitative Inquiry, Qualitative Research, Qualitative Research in Psychology, and Qualitative Sociology).

When questions arose from the material collected, additional literature from the broader field of qualitative research was searched. Extra information on the transcription of audio-recorded interviews conducted in the field of qualitative research was thus included, using the search terms “transcription”, “transcribe” and “note-taking”, combined with “qualitative research”. Additional literature was also searched regarding the role of insider-researchers-specifically nursing professionals-performing qualitative research. The following search terms were used: “insider researcher”, “nurse researcher”, and “researcher-researched relationship” combined with “qualitative research”.

Practical, functional and applicable information about the principles and techniques of narrative inquiry was primarily derived from the literature for the development of a quality instrument. The three authors discussed their first interpretations and emerging findings on a regular basis to develop plausible interpretations. All three authors were familiar and experienced with the qualitative research methodology. The discussions encouraged reflection upon, and a consideration of, possible explanations and interpretations as they emerged in relation to the data.

## 3. Results

The result section of this article comprises two sections. The first section synthesizes and discusses the main theoretical principles derived from the literature. These principles include the contextualized and situated nature of a narrative interview, the open style of interviewing, the types of recording and transcription available, and the main types of analysis in narrative research. Insights into the role of care professionals as insider researchers are discussed. The second part of the results presents a schematic overview of the main design elements from the literature that are used as a theoretical basis for the development of a narrative quality instrument. This newly developed narrative quality instrument is named “The story as a quality instrument”.

### 3.1. Theoretical Principles from the Literature

#### 3.1.1. Interview as Contextualized and Situated

Narrative inquiry draws on the constructivist paradigm, with its phenomenological and hermeneutic foundations and the idea that interpretative processes are subjective and culturally rooted. Narrative inquiry is also founded in the poststructuralist paradigm, in which the social reality is viewed as constructed and fluid and the use of language as transparent medium is problematized [22,23]. Post-modernism and post-structuralism called into question the researcher’s authority in knowing or asserting knowledge for the reason that an objective reality is challenged. The position of the researcher and respondent is reconceptualized as bounded to a specific context, situation, and time. Narrative researchers embrace the particular experiences of actors to understand them within specific places at specific times [24,25]. The interview is a discourse which is jointly constructed between the researcher and the respondent [26]. Narratives are therefore interactively produced and interpreted in the interaction between researcher and respondent. At the same time, creating narratives also constitutes to someone’s identity and experiences [18].

Furthermore, language is no longer perceived as value-neutral. People may not hear a sentence through the same meaning-frame and may therefore understand words differently than was intended by the speaker [23]. Each word has so many meanings that we cannot say only what we intend to say but unconsciously say more [24]. The meanings of interview questions and answers are not fixed but instead emerge, develop, are shaped by, and in turn shape, the discourse [26]. In qualitative research, words spoken by respondents will be edited and filtered through the lens of the researcher and their framework: The researcher hears the meanings of words through their own knowledge framework. It is therefore important to place the research content, the researcher, and the analysis process within the social and cultural context [27]. Although each interview is always inevitably a collaborative construction, the authentic voice of the narrator should be at the forefront [28].

In summary, the influences of constructivism, post-structuralism, and post-modernism led to the conception that a (narrative) interview is bounded to the specific context, situation, and time in which it takes place. Contextual information can thus enrich the interpretation of interview data. The stories shared by a respondent are also filtered through the interpretation lens of the researcher, and therefore, the background and meaning-frame of the researcher are inherently part of the research process and should be made explicit.

#### 3.1.2. Using an Open Style of Interviewing

One major pitfall in conventional qualitative research is to ask “sociological questions” instead of simple questions related to life experiences, which results in brief reports instead of detailed and longer stories [26,29]. Instead, one of the unique characteristics of the narrative methodology is its focus on an open interview approach, in which the interviewee feels free to express themselves [30]. A question-response structure is avoided, and the responses of the interviewee are not restructured or reformulated, in order to minimalize the influence of the interviewer [12]. In this way, the interviewer enables the interviewee to tell their story in his own way [31]. The interview agenda is open to input and change depending on the interviewee’s experiences [32]. In conventional forms of interviewing, the narrative flow of a respondent is often interrupted by questions from the interviewer. In narrative interviews, the respondents should instead be enabled to talk freely and spontaneously about their experiences [27]. An open, non-directive question encourages the natural flow of telling and invites the respondent to speak from a temporal account [22].

An open narrative interviewing style will elicit experiences of the whole personhood, rather than smaller accounts of, for example, illness [15]. Interpretation and content should be based on the interviewee’s perspectives throughout the whole narrative research process, including by using the person’s own words [30]. Such inductive narratives open up fresh and humane ways of accessing the life worlds of clients [11].

The role of the interviewer is to actively listen and facilitate the interviewee in sharing their story by encouraging them *verbally*, by humming, affirmative statements, or asking clarifying questions; and *non-verbally* for example, by nodding, facial expressions and adopting a position of attentive listening [12]. Only when an interviewer listens non-judgmentally and there is mutual trust, the respondent will be able to express themselves freely [30]. The interviewer should therefore follow ethics such as respect, mutuality, and openness to multiple voices and negotiation [24].

Fritz Schütze and Gabriele Rosenthal developed with their colleagues from the German Bielefeld Sociologists’ Working Group a clear method for open narrative interviewing within biographical research to present a whole life story regardless of the thematic focus [33,34]. This method, which is used widely nowadays, was developed originally to study the lives of holocaust survivors and Nazi soldiers [33,34,35]. When people are invited to tell their story at length and in their own way, they reveal what is important to them and unfold the “gestalt” informing their life. The open narrative interview method consists of two phases. In the first phase, the interviewer poses one single, open, initial question which is also an invitation: “Please, tell me your story”, which can be related to a particular topic. Thereafter, the interviewer should not interrupt the narration but maintains eye contact and makes encouraging sounds and comments. The researcher may note down some key words to remember questions that can be asked later on. In the second phase, the interviewer first poses internal questions on subjects that have been mentioned by the respondent, following the thematic structure of the story. Additionally, some external questions can be posed about topics which have not been mentioned yet. At all times, questions asking for an opinion or a reason should be avoided. In short, four principles are leading in the open narrative interview: (a) using open-ended questions, the more open the better; (b) eliciting detailed and particular stories; (c) avoiding “why” questions, as these interrupt the flow of the interviewee; (d) following up on the respondent’s wording, ordering and phrasing to retain their framework of meaning [23,33,34,35].

This type of open narrative interviewing was used as the basis for the development of two closely related narrative methods: the biographic-narrative interpretive method (BNIM) and the free association narrative interviewing (FANI) technique. Similar to the original method, BNIM uses a single question aimed at inducing narrative: a minimalist-passive narrative-inducing question with no further interruption of the respondent resulting in multiple particular incident narratives within a long narration [36,37,38]. Optionally one or two sub-sessions can follow depending on the nature of the research, in which probing questions arising from the first session can be asked [36,37,38]. Hollway and Jefferson (2001; 2012) adapted the open narrative interviewing method in their free association narrative interviewing technique (FANI). On the basis of psychoanalysis, Hollway and Jefferson look for social and psychological explanations of conscious and unconscious behavior about which the respondents tell a deeper story of the underlying emotional dynamics and formative experiences. Free associations are used to elicit deeper meanings and underlying explanations, through both the contents of the story told, and on the relationship established between the respondent and the researcher [39,40,41].

In summary, an open narrative interview structure based on one open invitation is proposed in narrative literature to invite respondents to talk about their experiences freely. The role of the interviewer is to listen and encourage the respondent to elaborate further. After the first part of the interview in which the interviewer interferes as little as possible, a second part can follow in which additional questions for clarification, concrete examples and additional topics can be posed.

#### 3.1.3. Transcription

There are several ways to recount or record qualitative data with regard to (narrative) interviews, such as field notes, transcripts of digital audio-files, and working directly from digital files [42]. Transcription refers to “the process of reproducing spoken words, such as from an audiotaped interview, into written text” [43]. Transcripts are described as having some advantages over field notes, as field notes are less precise and detailed, with the risk of simplistic interpretation. An interview transcript is more complete and more reliable as the interview can be analyzed in more detail, by multiple researchers [42] and the transcription process can deepen insights and understanding [44]. Any transcription of speech must be seen as a compromise as intonations, emphasis, and non-verbal behavior will be lost in transcription [27]. Some qualitative researchers therefore choose to work directly from digital audio files to avoid misrepresentation and loss of context, as emotional content, intonation, laughter, and silences are best captured in this way [42,45].

When the choice for transcription is made, a detailed transcription of the recorded narration is used for narrative analysis [30]. The researcher will write out the whole interview, by typing out both the respondent’s words and those they used themselves to capture the entire interview, including emotions and notable non-verbal signals [30]. *Verbatim transcription* is a well-known form of transcription and refers to “the process of reproducing spoken words, such as those from an audiotaped interview, into written text” [43]. A “clean” or “verbatim” transcript makes the content of the material easy for non-scientific or lay readers to read, although no extra information is included on the form. It captures the chronology of events and evaluative elements in the precise words chosen by the interviewee [27].

More detailed types of transcription include additional information on the speaker’s use of intonation, pauses, rhythm, hesitations and body language, depending on particular analytical interests [27]. For example, transcripts produced for conversation analysis can include additional description and symbols to indicate body language such as frowning or gasping, interruptions, the prolongation of sounds, and intonations [27,46]. The unit of discourse approach by Gee is another example, in which the rhythm and structure of speech are included by splitting the transcript into groups of short lines often ending with a fall in voice or a short pause [47,48]. It is less time consuming to create and read the verbatim transcript compared to more advanced types of transcriptions [27]. The amount of detail needed for the transcription can be determined by bearing in mind the aims of the research [49].

In summary, digital audio recordings are often used and transcribed into written text afterwards, which is considered a thorough and reliable procedure in qualitative research. Verbatim transcription is often chosen as a compromise to balance scientific rigor with the amount of time and scientific abilities required, when the analysis is primarily focused on the content rather than structural components of the narrative (see Section 3.1.4. for both options).

#### 3.1.4. Different Types of Analyzing Narratives

**Figure 1:**
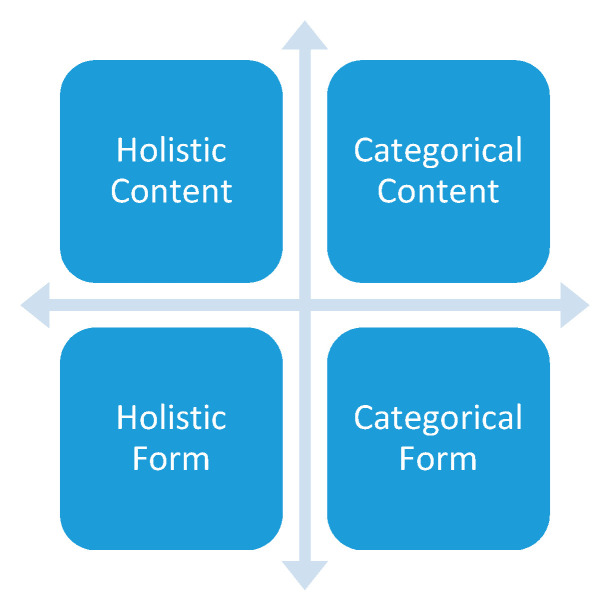
Dimensions of analyzing narratives [48].

Narratives can be analyzed in different ways, in two main dimensions: (a) thematic versus holistic approaches and (b) content versus form (see Figure 1) [50]. Polkinghorne (1995) describes the first dimension as distinguishing “the analysis of narratives” and “narrative analysis” [51].

- Categorical Content

The *analysis of narratives* involves a thematic and paradigmatic type of narrative inquiry in which stories are collected as data to analyze the descriptions of common themes or categories across the stories, characters, or settings (Figure 1: Categorical Content). This type of analysis functions to generate general themes from a set of narratives and moves from the stories to common elements [51]. The narratives are primarily seen as the data and thus the starting point of the analysis [52]. This analytical approach can be chosen when a researcher is primarily interested in a certain problem shared by a group of people [50]. Within this categorical-content type of analysis, a researcher can either choose to derive the concepts inductively from the data or take a more theoretical position by deriving concepts from previous theories and determining whether these concepts can also be found in the data. The grounded theory method of Glaser and Strauss (1967) is often used when a more theoretical approach is chosen [51,53].

- Holistic Content

The second type of analysis involves the holistic *narrative analysis* (Figure 1: Holistic Content). Descriptions of events and experiences are collected and synthesized into a story that unites and gives meaning to the data. Narrative analysis thus moves from elements to stories. Where a categorical content analysis separates the narrative in themes, the second type of analysis synthesizes the data into a story [51]. The narrative is seen as the product and end point of the analysis [52]. The researcher discovers a plot that connects crucial events and other data elements and composes these elements into a whole or story [51]. Sections of the narrative are holistically interpreted in the context of other parts. This type of analysis is chosen when there is a focus on each person holistically, and the developments of each individual towards the current position, simultaneously embracing the variations among people [50]. Holistic analysis is widely used in narrative research, and the procedures are described in detail in Section 3.1.4.1.

- Holistic Form and Categorical Form

Where the first two types of analysis are common in that they are focused on the content of what is told (what, who, why, how), another type of analysis focuses primarily on the form: the structure and progression of the plot, the sequence of the events, the choice of metaphors and phrasing of words. The form can be analyzed both holistically and categorically [50]. The form is harder to manipulate by the respondent and so implicitly elicit deeper layers of the respondent’s identity. A speaker can be unaware of, or deny something verbally, but hidden emotions come forward based on an analysis of form [50]. In a well-known example, Labov and Waletzky identified formal structural properties which often recur in narratives and can be used to analyze each element: the abstract, orientation (time, place, situation, and participants), complicating action (what happened), the evaluation (by the narrator), the resolution (how it ended), and the coda (returning to the present) [27,54]. Another example focuses on sections of narratives and analyzes the formulation and repetition of phrases indicating degrees of criticism of respondents to their relatives or the emotional disturbance surrounding certain events [50]. Examples in the literature show that substantial analysis skills and abstract interpretation levels are required to conduct such structural types of narrative analysis [50].

##### 3.1.4.1. Holistic Content Analysis: Restorying

In (holistic) narrative analysis, researchers analyze transcripts and field notes and consequently *retell* or *restory* the narratives of the research participants. This process is appropriately called “*restorying*” in narrative research: the process of collecting stories, analyzing them for key elements and then composing and rewriting each story in chronological sequence [55,56]. This creates the difference between first-order narratives–the stories that individuals tell about themselves and their own experiences–and second-order narratives–which researchers construct to make sense of the experiences of respondents [27]. Rich detail is included about the setting or context in which the experiences of the respondent physically take place [55]. Restorying involves developing a plot-what the story is about-by linking the data elements into a meaningful whole [51]. The story plot retrospectively aligns events and actions to make sense of experience [57].

The general eclectic methodological approach *portraiture* is closely linked to restorying but originates from a blending of qualitative methodologies such as life history, naturalist inquiry, and ethnographic methods [58]. Similar to a visual artistic portrait, portraiture captures and represents the research participants and their experiences through the critical lens of the researcher [59]. It shows a respondent’s experiences holistically and moves beyond the walls of the academia by communicating people’s experiences in understandable language [59,60].

By analyzing, interpreting, and reordering a story, it is unavoidable that the researchers voice is included in the text produced [61]. To remain close to the voices of the participants, Byrne (2015) used a participant’s words and used writing in the first person from the perspective of the respondent who shared the story [61]. This kind of restorying, in which researchers base their reports on the participant story and wording, is also called the inductive mode [22]. Another effective measure to guarantee the voices of the participants are represented well in the final story is *member checking* [28]. It is crucial that the researcher reflects on their framework prior to entering the field, including norms, values, and ideological and autobiographical assumptions [59].

An author can choose to present a narrative from the first, second or third person; and the perspective from which the story is told (respondent or researcher). An advantage of the first person respondent position is that the reader sees the story from the perspective of the participant, which increases feelings of affinity. It also limits the potential to include information or reflection from other viewpoints, however, such as that of the researcher [62].

Hollway and Jefferson (2001) provide an example of restorying from the free association narrative interviewing technique they developed. After an interview, they drafted a “pen portrait” aimed to make the person come alive to the reader. A pen portrait is largely descriptive, around five pages long, and provides a great deal of information. A two-page form was completed for every respondent, containing standard biodata (age, sex, race, marital status, family, and health) and comments on the themes that emerged [39]. Another example of a procedure for restorying is the step-by-step guide of Murray and Sools, in which the analysis focusing on the storyline of the narrative is separated from three other stages: the interactional analysis focused on the positioning of the storyteller, the contextual analysis, and a comparative analysis between cases [63].

In short, narratives can be analyzed holistically or categorically, with a primary focus on either the content or the form. In reality the distinctions are less rigid, as researchers use combinations of different types of analysis. When a study is focused on each individual and focused on the content of narratives, the holistic content analysis is the most legitimate approach. A portrait or second-order narrative is composed of the key elements, often chronologically restructured. The whole is seen as a sum of its parts, and rich detail is included. In an inductive mode, the wording of the respondent can be followed to remain close to the voice of the respondent.

#### 3.1.5. From Theory to Quality Assessment Research: The Role of a Care Professional as Interviewer

A narrative interview is always a joint product of both the participant, as data supplier, and the researcher, as data collector and analyst [14]. The participant interprets their experiences by telling a story in an interview, and when the researcher analyses the stories told, they make a new interpretation, a new story [13]. The interviewer is also involved in the interaction and the questions posed to the interviewee. So the interviewer both shapes and is shaped by the context they study [24]. Why, to whom, and how the story is been told determines what the story is about [64]. A researcher’s inherent subjectivities matter in an interview, including background characteristics, values, beliefs, and emotions. Rather than treating the interviewer as an objectified neutral party in the interview, the influence of the researcher’s personality should be accepted as centrally involved in the research process, and therefore made explicit [65].

It is argued that there are both advantages and disadvantages to being an insider-researcher [66]. Advantages include speaking the same language, understanding local values and knowledge, and knowing the formal and informal power structure [66]. Insider researchers from the nursing professions benefit from social skills [67,68,69,70], reflection abilities [68], and their more equal position with clients, compared to academic clinicians, psychiatrists and perhaps academic researchers [68].

Disadvantages related to the insider-position might include the role duality, an assumed knowledge of the participant’s views, and overlooking certain routine behaviors [66,71]. Several measures should be taken to prevent role confusion or a blurring of role boundaries between the insider researcher and the respondents [65]. Measures include clearly defining the interviewing role and noting professional background [70,72], not wearing a clinical uniform [70], and providing a transparent explanation of confidentiality issues, including specifying the persons with whom the data will be shared [71].

Care professionals are discouraged from interviewing their own clients as they might feel forced to participate and it might prompt clients to give socially desirable answers due to their care dependence [72,73,74]. It is also advised that interviewers consider doing the research at a different site, but in a similar setting [71]. One nurse researcher favored the position of being both an insider and outsider by studying clients on another ward and noted that these unfamiliar clients seemed open and honest [68].

There might be dilemmas during interviews when a care professional is asked to intervene or provide assistance [67,72]. Care professionals must decide how to respond if intervention is not immediately needed: (a) step out of the researcher role and intervene or (b) remain in the researcher role and refer the client to professionals elsewhere [67]. If direct intervention is needed, the care professional should document the event and the reasons for intervening [72]. To minimize ethical dilemmas related to role diffusion, care professionals need to consciously reflect on their dual roles when interviewing [68]. The interviewer has to truly understand and internalize the purpose and value of being a researcher and the research process [75]. Role play could help researchers be better prepared for dilemmas and similar situations, and discussion with colleagues during the data collection can be beneficial [76].

In summary, care professionals are habituated in specific care contexts and can therefore be defined as “insider researchers”. Their insider position provides both advantages and certain challenges. Care professionals taking on the role of insider researcher are therefore discouraged from interviewing their own clients and encouraged to make both their professional background and their role as interviewer explicit. Role play and discussion with colleagues can be beneficial in concretizing ways to deal with ethical dilemmas.

### 3.2. Development of a Narrative Quality Instrument

Based on the literature synthesized in the first section of the results, a new quality instrument is developed to collect narratives from older adults receiving long-term care. Table 1 summarizes the main design principles derived from the literature, which are included in the new narrative quality instrument.

Care professionals will take on the role of insider researchers and interview older adults with whom they do not have a care relationship. Care professionals receive a training to be prepared for performing the interviewing role, such as practicing narrative interviewing skills and analyzing the narratives. The open narrative interviewing method is followed to allow older adults to talk freely. After the open invitation “You have been receiving care at organization X for a while. Please tell me about this.” the flow of a natural conversation is followed. The interviewer will be instructed to not introduce any further themes but to keep the conversation going so that it follows the flow of a natural conversation. Non-verbal body language and verbal cues from the wording and phrasing of the respondent will be used to invite them to continue speaking.

When the older adult seems to have finished their story, the interview moves into the second stage. In the second part of the interview, probing questions can be posed to supplement information shared by the older adult using their own wording. Some probing questions will be tailored specifically towards care provision and possible areas for improvement to make the narratives suitable for quality research. Interviews are audio-recorded and transcribed verbatim afterwards, and used to create a holistic portrait of each interviewed older adult.

As the narrative method can elicit the individual and diverse experiences of older adults from their position in the lifeworld, narratives can provide a rich description from the older adult’s perspective on their own life and, specifically, the care provided. Care professionals can use the newly developed instrument to discover and collect the experiences of older adults receiving long-term care from an inductive perspective following the narrative methodology. The collected narratives can be used for a variety of purposes, including team reflection to achieve improvement in quality towards person-centered care. A detailed description of the instrument “The story as a quality instrument” is included in the Appendix A.

## 4. Discussion

There is a need in Dutch long-term care practice for older adults for a scientifically substantiated qualitative instrument to evaluate client experiences of the care provided from an insider’s perspective [9,10,77]. Narratives can provide a rich description of an older adult’s life from their own point of view and, specifically, the care provided [11]. Using a narrative approach means that older adults are likely to talk in a natural and unstructured way about the events that matter to them most [16,17]. In the current study, a theoretical approach was used to develop and substantiate a practical narrative quality instrument to discover the experiences of older adults receiving long-term care. “The story as a quality instrument” was developed to collect narratives for quality research based on the theoretical principles and techniques of narrative research. Care professionals will take on the role of insider researchers and interview older adults with whom they do not have a care relationship. Care professionals receive training to prepare them for performing the interviewing role, for example, practicing narrative interviewing skills and analyzing the narratives. The open narrative interviewing method is followed to allow older adults to talk freely. After one open invitation, the flow of a natural conversation is followed. In the second part of the interview, probing questions can be posed to supplement information. Interviews are audio-recorded and transcribed verbatim afterwards and used to create a holistic portrait of each interviewed older adult.

There are issues related to the future implementation of the developed instrument, which must be explored in more depth. Prior to the execution of the instrument, the care organization needs to determine the goal(s) towards which the quality instrument will be deployed. In addition to quantitative forms of quality measurement for external accountability, this instrument can be used to uncover relevant insights into care provision. The narratives can be used for quality research on an *individual level*, for a whole *location or team,* or even a whole *care organization*. In the second or third case, the portraits can be used by care professionals to determine relevant themes and areas for improvement in the care process and policy more generally, thus transcending individual care by formulating team goals, proposing follow-up actions, and making policy changes. This has implications for information provision prior to the interviews and the degree of comfort with which clients will share their experiences. When the instrument is used to improve the individual care of the clients interviewed, the portraits will not remain anonymous for the care professionals who are going to work on the issues. Clients might therefore be less open and more careful in what they share, fearing negative consequences for care relationships or the care provided. Older adults in particular find it hard to share (negative) feedback and tend to avoid complaining [78]. When narratives are used for quality research at the team or location level, identifying information such as names and revealing hobbies or interests can be removed. Clients might feel more able to speak freely and share negative experiences when confidentiality is strived for. Complete anonymity might still not be possible due to the uniqueness of individual stories, and therefore, it is important to discuss with respondents which target audiences will use the stories for what purpose and ask for consent [79].

Another issue regarding implementation concerns which care professionals can be invited as insider researchers applying the quality instrument. A variety of long-term care professions focus on older adults, including care aides, nurses, and occupational therapists organizing daily activities. The focus and content of the required education differs between these professions, from more practical training to more academic and theoretical orientation. In addition to formal education, certain individuals might fit better with narrative interviewing based on their personality, interests, and capabilities. Some basic interview and analyzing skills are required for proper application of the instrument. Adopting an open and inviting approach so as not to steer older adults in a certain direction is important to enable the interviewees to speak freely and to ensure sufficient quality of the narrative information. Some analytic skills and writing abilities are minimally necessary to develop a portrait which is non-judgmental or prejudiced and covers the relevant information shared. The training aims to create opportunities for practicing and developing these required skills, for example, by providing detailed instructions for the data collection procedures and by scheduling trial exercises with colleagues. Other measures taken to guarantee the quality of the data collection process include peer reflection during the quality research and analyzing the same narrative in pairs to learn from the different viewpoints. It is still possible that some individuals or specific professions are better equipped to interview or create a portrait than others.

Finally, one of the conditions for the proper execution of the instrument concerns sufficient time available to attend the training and undertake and analyze the interviews. When care professionals play a central role in quality research, it gives them extra responsibility in their—already busy—schedule. Undertaking the training and three narrative interviews will take around 25 h of work, if the transcription is outsourced to a transcription agency. The quality framework for nursing home care encourages an atmosphere in which continuous learning and team development are central [10]. At the same time, several studies note the tight work schedule and high work pressure in nursing home care [80,81,82]. Future implementation will demonstrate whether the costs of the instrument balance against the benefits for the care professionals and care organizations using the instrument.

### 4.1. Strengths and Limitations

The study has some strengths and limitations. One strength concerns the theoretical approach used to develop a quality instrument to ensure a thorough and valid procedure for data collection. A substantial number of qualitative quality instruments are more pragmatically developed using a practical approach without a theoretical foundation without evidence regarding validity, reliability, and user experiences [19]. The study resulted in a relatively straightforward instrument which can be applied in practice for a variety of purposes, from the micro-care process to team learning and bottom-up innovation at the location level. The instrument was developed in order to interview older adults who are physically and mentally capable of describing their care experiences themselves. An observational method might be more suitable for older adults with non-congenital brain damage, mental decline, or physical conditions hindering them from talking about their experiences or, alternatively, an instrument focused on the experiences of relatives.

The manual search procedure is a drawback of the study design. Although the search procedure, including search terms and the focus of the review, was described in detail in the method section, it was carried out manually, meaning that some relevant studies might have been missed which would have been included with a more systematic search procedure. The included literature was not assessed on quality criteria, which could have provided the reader with more insight into its methodological quality.

### 4.2. Future Research

Several topics for future work arise from this study. A systematic procedure for narrative data collection and analysis was developed based on the academic literature. The current study did not include empirical testing of the instrument developed. A follow-up study evaluating the empirically testing and execution of the instrument will provide more insights into the feasibility and usability of the instrument in practice. Another necessary next step for the appropriate use of the portraits concerns the development of an approach to actually use the narrative portraits for team reflection to achieve quality improvement. Action is the missing link in the literature, as client experiences should not only be heard but also acted upon [20]. This requires a systematic approach to use client information for quality improvement efforts across teams and the organization [20]. This approach can best be designed in co-creation with stakeholders (e.g., care professionals, quality employees, and client representatives), to ensure that their needs and conditions are taken into account [83,84]. Finally, whether the instrument can be used in other settings (e.g., in the curriculum of studies for future care professionals) and for other client groups receiving care could be explored.

## 5. Conclusions

A theoretical approach was adopted in this study to develop and substantiate the narrative quality instrument “The story as a quality instrument”. The open narrative interviewing method is selected for structuring the interview, followed by a holistic narrative analysis resulting in individual portraits of older adults. The instrument is deemed promising for practice to collect the narratives of older adults receiving care in a thorough manner, as the instrument is composed of theoretical principles and techniques of narrative research. Empirically testing the feasibility and usability of the instrument is the next step in development. A systematic approach must be taken in the future to translate narrative portraits into actions targeting quality improvement in the long-term care of older adults.

## Figures and Tables

**Table 1:** Overview of the design principles used for the narrative quality instrument.

**Design Elements**
**The Interview**
Avoid a fixed question-answer structure or “sociological questions” to enable an interviewee to tell their story in their own way. An open narrative interviewing method is selected for structuring the interview: (1) Pose one single open initial invitation at the start of the narrative interview to induce client experiences inductively, for example “Please tell me your story”. (2) Follow the four basic principles to elicit holistic experiences from the client’s perspective: - Use open-ended questions, the more open the better; - Elicit detailed and particular stories; - Avoid “why” questions as these interrupt the flow of the interviewee; - Follow up in the wording, ordering, and phrasing of the respondent to retain the meaning frame of the interviewee. (3) In the second part of the interview, additional internal questions for clarification, extra information, and examples can be posed that arise from the first part. The order and wording of the respondent are followed. Additionally, some external probing questions can be posed related to topics that were not yet discussed. In the first part of the interview session, the interviewer interferes as little as possible, limited to confirmatory actions such as eye contact and encouraging comments to maintain the gestalt of the respondent’s story.
**Procedure to record data**
Make an audio-recording of the interview, and transcribe the interview *verbatim* afterwards to recall and analyze the interview findings accurately and easily. Verbatim transcription was chosen considering the more practical aim of the use of the narratives for quality research, as it is easier for care professionals to create and interpret and is less time consuming than more advanced transcription options.
**Analysis and reporting**
A holistic narrative analysis focused on the interview content is followed to produce a portrait of the experiences of each individual older adult, written in understandable language. Read the interview transcript, search for the key elements, and compose and rewrite the story in a chronological sequence. This results in a portrait which includes rich detail on the shared perceptions, events, setting, and context. Use the wording and phrasing of the respondent and an inductive mode to remain close to the client’s voice. Optionally, member checking of the portrait can be used to guarantee a reliable representation of the narrative from the client perspective. The portrait can be written in either the first or third person (a) based on personal preference and (b) depending on the amount of information the researcher wants to add from their own perspective. If a researcher wants to share information from their own perspective, the portrait can be written from a third person perspective, as information from their own perspective cannot be included in the first person.
**Central role of care professional as insider researcher**
According to paradigmatic views influenced by constructivism, poststructuralism, and post-modernism, each interview is bound to the specific context, situation, and time in which it takes place. The words spoken by respondents will be edited and filtered through the lens of the researcher and their framework. The researcher should therefore make their background and personality explicit, as these are centrally involved in the research process. Care professionals who take on the role of insider researchers should counteract role ambiguity by defining their interviewer role and mentioning their professional background to clients. Care professionals are discouraged from interviewing their own clients and should ideally not wear a clinical uniform during the interview. Reflection with colleagues and role play in the research preparation are proposed in order to adequately deal with ethical dilemmas related to the position of insider researcher. Care professionals should inform the respondents of the purpose of the narrative interview, the type of recounting or recording used, the confidentiality measures, and the target audiences with whom the findings will be shared.

## Data Availability

Not applicable.

## Appendix A. Description of “The Story as a Quality Instrument”

This section describes the characteristics of the instrument “The story as a quality instrument” in detail. Care professionals will be appointed as insider researchers to achieve individual and collective learning, increase understanding of client perspectives, benefit from their contextual knowledge, and gain support for the measures taken in the quality research. Following the advice of previous insider researchers, care professionals will be instructed to interview older adults with whom they do not have a care relationship [72,73,74] and who are ideally clients from a different team [68,71]. This will create a safe interview environment and reduce the likelihood that older adults will give socially desirable answers.

The care professional will start the interview by introducing themselves and their professional background, and highlighting their interviewing role [70,72]. They will explain that the respondent can talk about their experiences freely. The confidentiality of the conversation and the use of the information will be explained, for example, that identifiable information such as the respondent’s name and the names of anyone mentioned will be removed from the transcript [71]. The reason for audio-recording will be explained. The interviewer will offer the opportunity to ask any questions and will ask the older adult to sign an informed consent statement to confirm their willingness to be interviewed. The narrative interview can then formally start.

The open interview approach of the biographical narrative interviewing method is used as the main foundation for the interview process [23,35]. To allow the older adult to talk about their experiences freely, the interview will be based on one simple open invitation: “You have been receiving care at organization X for a while. Please tell me about this.” The interviewer will be instructed to not introduce any further themes but to keep the conversation going so that it follows the flow of a natural conversation. Non-verbal body language such as an active listening position, using eye contact, nodding and humming will be used to assist and encourage the respondent to continue their story. Verbal cues from the wording and phrasing of the respondent will be used to invite them to continue speaking, for example, repeating the respondent’s last sentence, asking a small clarifying question or summarizing what has been said. When the older adult seems to have finished their story, the interview moves into the second stage. In the second part of the interview, probing questions can be posed to supplement information shared by the older adult using their own wording [23,35]. Some probing questions will be tailored specifically towards care provision and possible areas for improvement to make the narratives suitable for quality improvement.

Each interview will be audio-recorded to allow a verbatim transcription of the interview [30]. After the interview, the interviewer will note down any impressions or insights they had during the interview, in addition to the transcript, such as contextual elements, emotions, or body language [30]. Using the verbatim transcription, the care professional will analyze the transcript and “restory” the most important themes into a portrait [39,59]. The “portrait” is the analyzed story, so there is a shift of “what has been told” to “what the care professional has heard” [27]. A reliable representation of the respondent’s story will ideally be achieved by staying close to the respondent’s words, using literal transcription of recordings [61], and working in pairs on the first analysis to stimulate discussion [76]. A more inductive mode of restorying will be used [22]. Any characteristics of the respondent that make the portrait traceable will be removed or rephrased to make the portrait useful for quality improvement.

Training will be provided in three meetings to provide care professionals with the necessary background information and practice interviewing and analyzing skills to use the instrument. This will be provided by a trainer who continually shares feedback on exercises and stimulates care professionals to reflect on their experiences and their role as interviewers. The first training session consists of a combination of explanation and practicing narrative interview techniques. The explanation includes the meaning of quality of care, the difference between quantitative and qualitative methods, the basic theoretical principles of narrative inquiry, and the procedures of “the story as a quality instrument”. Role play is used to let care professionals practice the interview procedures [71]. After the first training session, the care professionals will carry out two narrative interviews. The second training session is primarily focused on reflection and peer discussion based on the narrative interview experiences [76]. Care professionals share their experiences with narrative interviewing, reflect on their role as interviewer, analyze the stories, and learn how to draft a portrait. After the second training session, the care professionals will carry out one narrative interview. In the third training session, the care professionals work further on their analysis of the portraits, share their experiences, discuss reliability and validity issues, and discuss the results of the three interviews.
